# Supplementary material for: Enhanced photocatalysis and biomolecular sensing with field-activated nanotube-nanoparticle templates
Source: Nat Commun. 2019 Jun 7;10:2496. doi: 10.1038/s41467-019-10393-9 (PMC6555825; doi:10.1038/s41467-019-10393-9)
Supplement: Supplementary file 1 — Supplementary Information [file 41467_2019_10393_MOESM1_ESM.pdf]

Supplementary Information for

Enhanced photocatalysis and biomolecular sensing with field-activated nanotube-nanoparticle templates

Sawsan Almohammed [a, b], Sebastian Tade Barwich [c], Andrew K. Mitchell * [a], Brian J. Rodriguez * [a, b], and James H. Rice * [a]

Corresponding Authors: james.rice@ucd.ie, andrew.mitchell@ucd.ie and brian.rodriguez@ucd.ie

Substrate fabrication and characterization

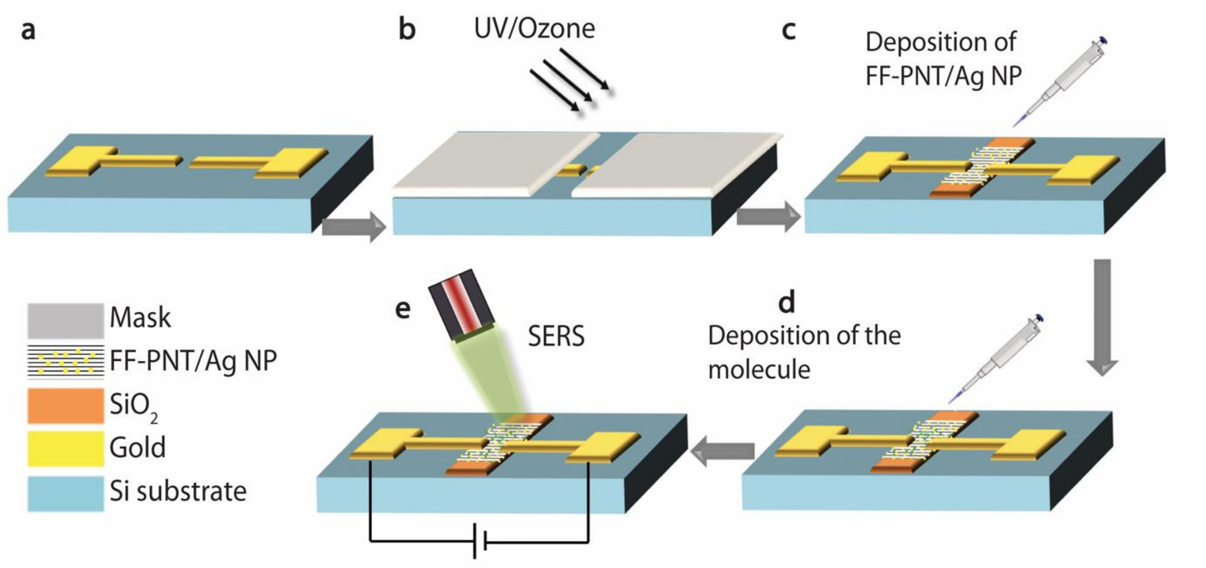

Supplementary Figure 1. Process to align FF-PNT/Ag NP template on the microfabricated gold electrode device. (a) Gold coating though 3d printed mask with ~0.1 mm opening size between electrodes. **(b)** Exposure to UV/ozone through physical mask with 1 mm gap to produce the insulating SiO_2 layer. **(c)** Deposition of FF-PNT/Ag NP solution onto the SiO_2 substrate (only) and drying. FF-PNTs are aligned on the SiO_2 substrate. **(d)** Deposition of the probe molecules on the aligned dried template. **(e)** SERS measurements on FF-PNT/Ag NP template with applied electric field.

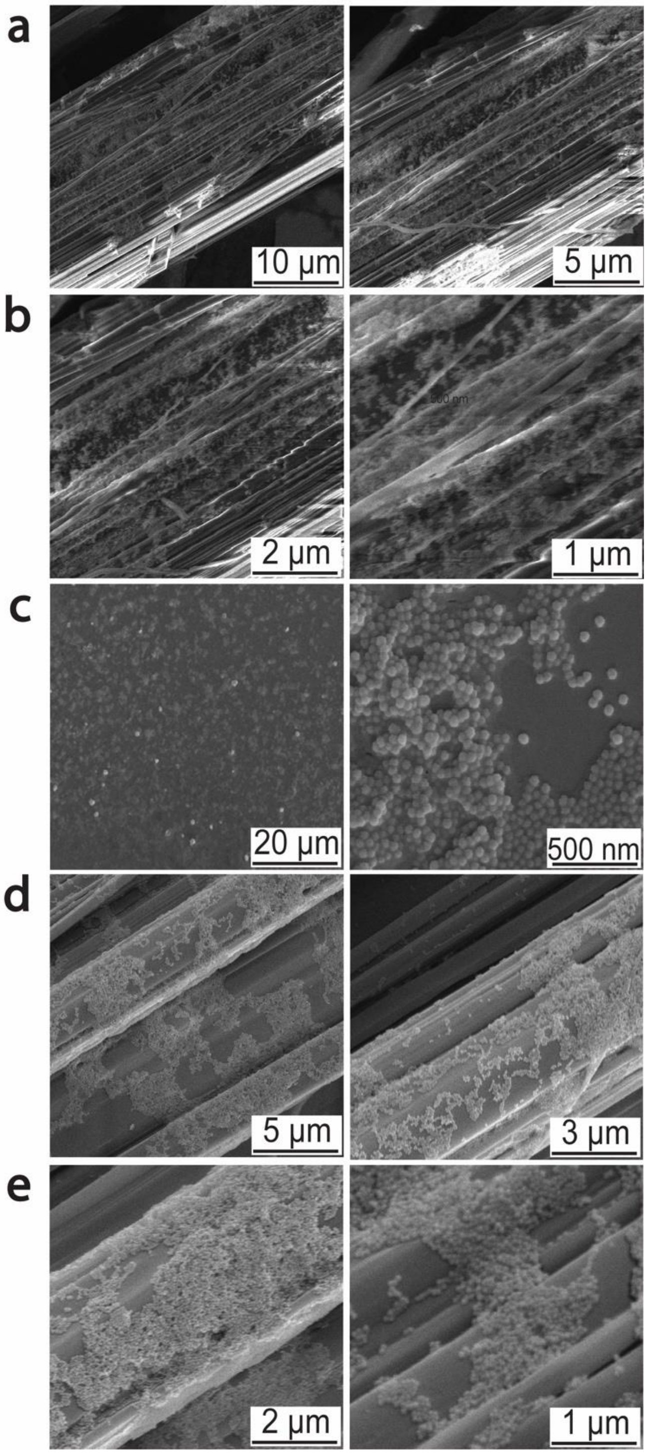

Supplementary Figure 2. Characterization of FF-PNT/Ag NP template. (a, b) Scanning electron microscopy (SEM) images for the FF-PNT/Ag NP template without gold coating, showing spherical features assigned as Ag NPs, with clustering predominately above and between the FF-PNTs. **(c)** SEM images of Ag NPs on Si without FF-PNT, with gold coating. **(d, e)** SEM images of the template FF-PNT/Ag NP with gold coating. Note that there is no observed change in topology or morphology before and after application of the electric field.

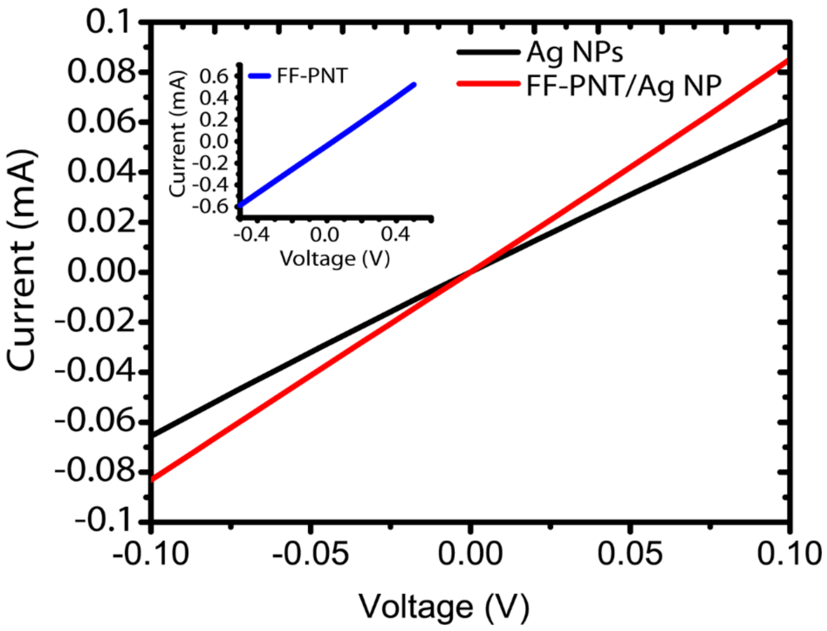

Supplementary Figure 3. Electrical conductivity studies on the template. IV (current and voltage) measurements of FF-PNT/Ag NP template (red), FF-PNT only (blue; inset), Ag NPs only (black). Data recoded using a Keithley device and a bespoke electrode substrate with dimensions 2 cm x 1 cm and gap of ~ 1 mm.

Reference SERS and Raman spectra for molecule PATP

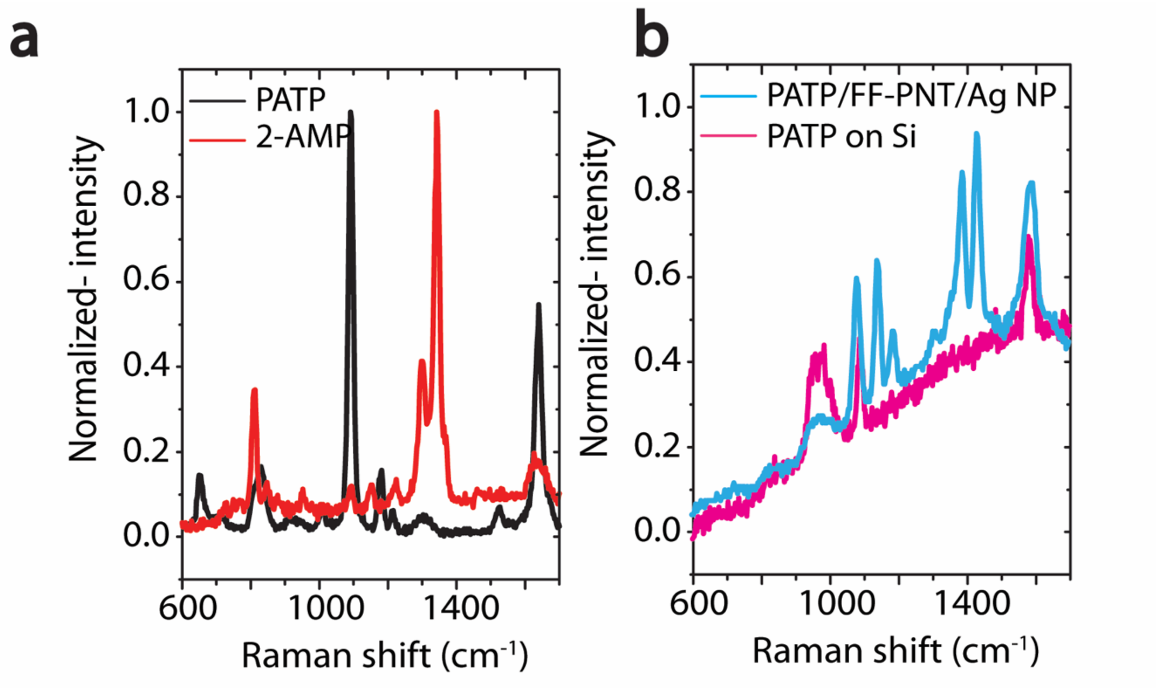

Supplementary Figure 4. Raman spectra of PATP. (a) Raman spectra of PATP and 2-AMP in powder form. **(b)** SERS spectra of PATP on the FF-PNT/Ag NP template (blue) vs on Si substrate only (pink). The SERS spectra for PATP is assigned to a combination of a- and b-type vibrational bands. Assignments of four of the five most intense bands (at 1142, 1390, 1433, and 1575 cm^{-1}) can be attributed to b_2 modes. The band at 1082 cm^{-1} can be assigned to an a_1 mode. In contrast, the Raman spectrum for PATP in powder form shows peaks at 1081, 1182, 1489, and 1593 cm^{-1} that are assigned to a_1 type vibrations[1,2]. The observed b-type bands are intrinsic to PATP and are associated with a chemical enhancement mechanism[1,2]. The four non-totally symmetric b_2 modes are known to be selectively enhanced in SERS through Herzberg-Teller vibronic coupling[3]. However, spectral differences may also originate from a photochemical reaction of PATP to form p,p′-dimercaptoazobenzene (DMAB)[4–6] with the b_2 vibrations of N=N at 1433 and 1390 cm^{-1} and the 1142 cm^{-1} peak assigned as a C-N vibrational mode[4–6].

SERS with applied electric field for molecule PATP

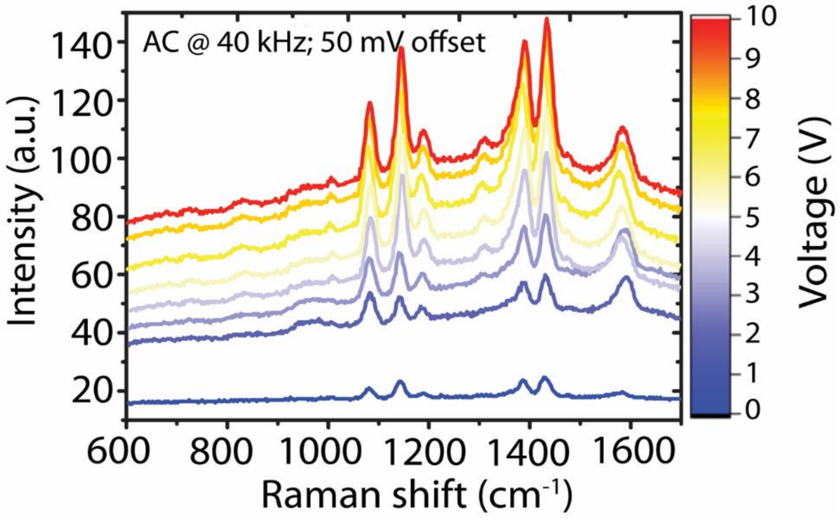

Supplementary Figure 5. **SERS measurements of PATP on the FF-PNT/Ag NP template.** SERS spectra are shown for PATP on the FF-PNT/Ag NP template at various voltages from 0 to 10 V at 40 kHz AC, with an offset DC bias of 50 mV. Increasing the voltage, while keeping the offset bias fixed at 50 mV, results in an increase in SERS signal intensity (up to ~7 fold at 10 V) with no change in the SERS spectral relative band intensities, indicating no oxidation product PNTP is produced.

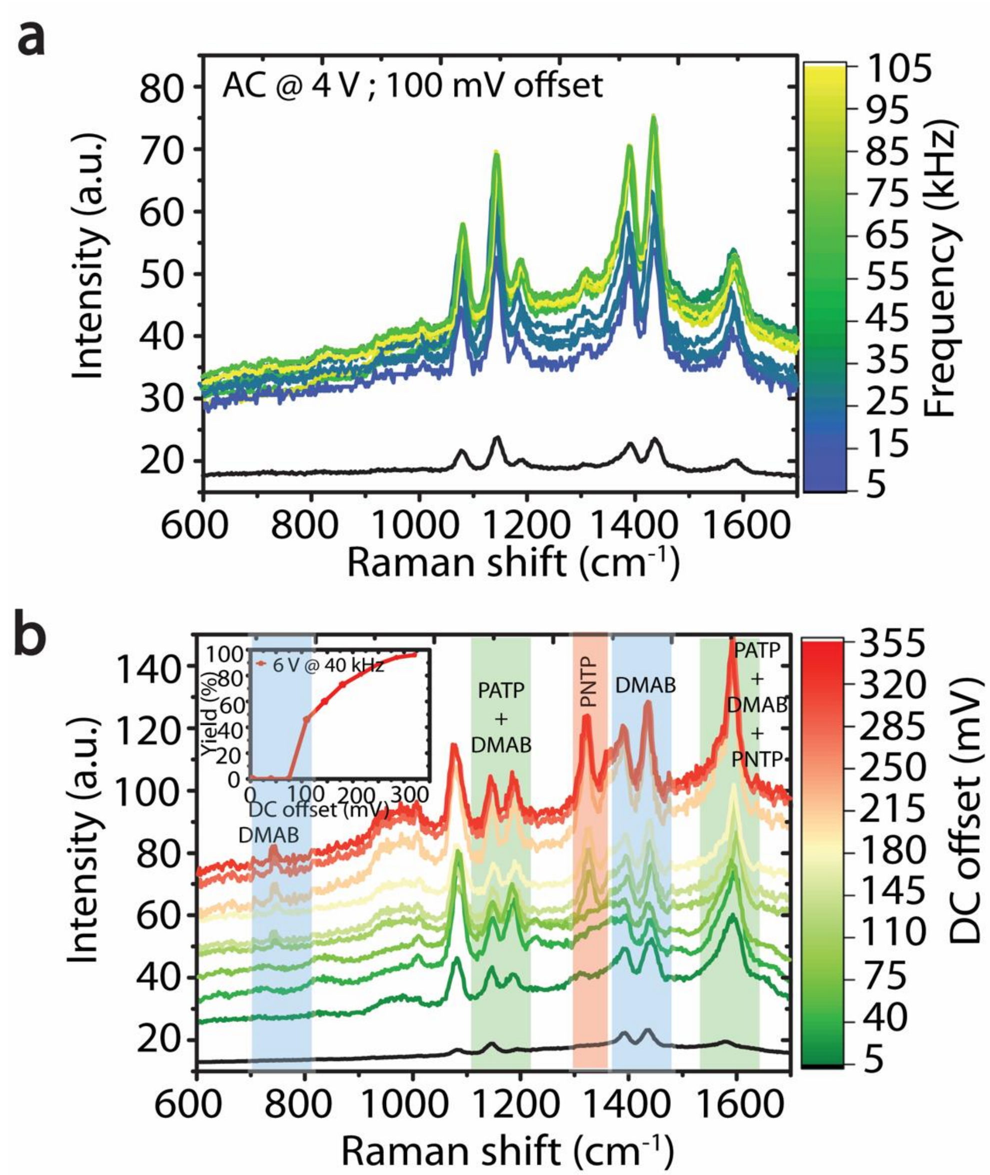

Supplementary Figure 6. SERS measurements of PATP on the FF-PNT/Ag NP template. (a) Data obtained at constant 4V and 100mV bias offset, varying AC frequency in the range 5-105 kHz. For reference, the black line corresponds to 0V. A ~7 fold increase in SERS intensity is observed, compared with the result at 0V (no electric field). We find that varying the frequency results in slight fluctuations in SERS intensity at high voltage and offset, which stabilizes at around ~40 kHz. Data presented in the main paper is therefore at 40 kHz. **(b)** SERS spectra recorded at different offset DC bias from 0-355 mV at a fixed 6V and 40 kHz AC. Inset shows the yield (formation of PNTP measured through the relative band intensity of the Raman mode at 1334 cm^{-1}) as a function of offset bias. Increasing the offset bias up to 355 mV resulted in an ~8 fold increase in SERS intensity. The SERS vibrational mode distribution also changes, with new vibrational modes at 1334 and 1376 cm^{-1}. These modes are assigned to symmetric NO_2 stretching vibrations of the product PNTP[4–6], created from PATP through a photochemical plasmonic-assisted catalytic oxidation reaction[4–7].

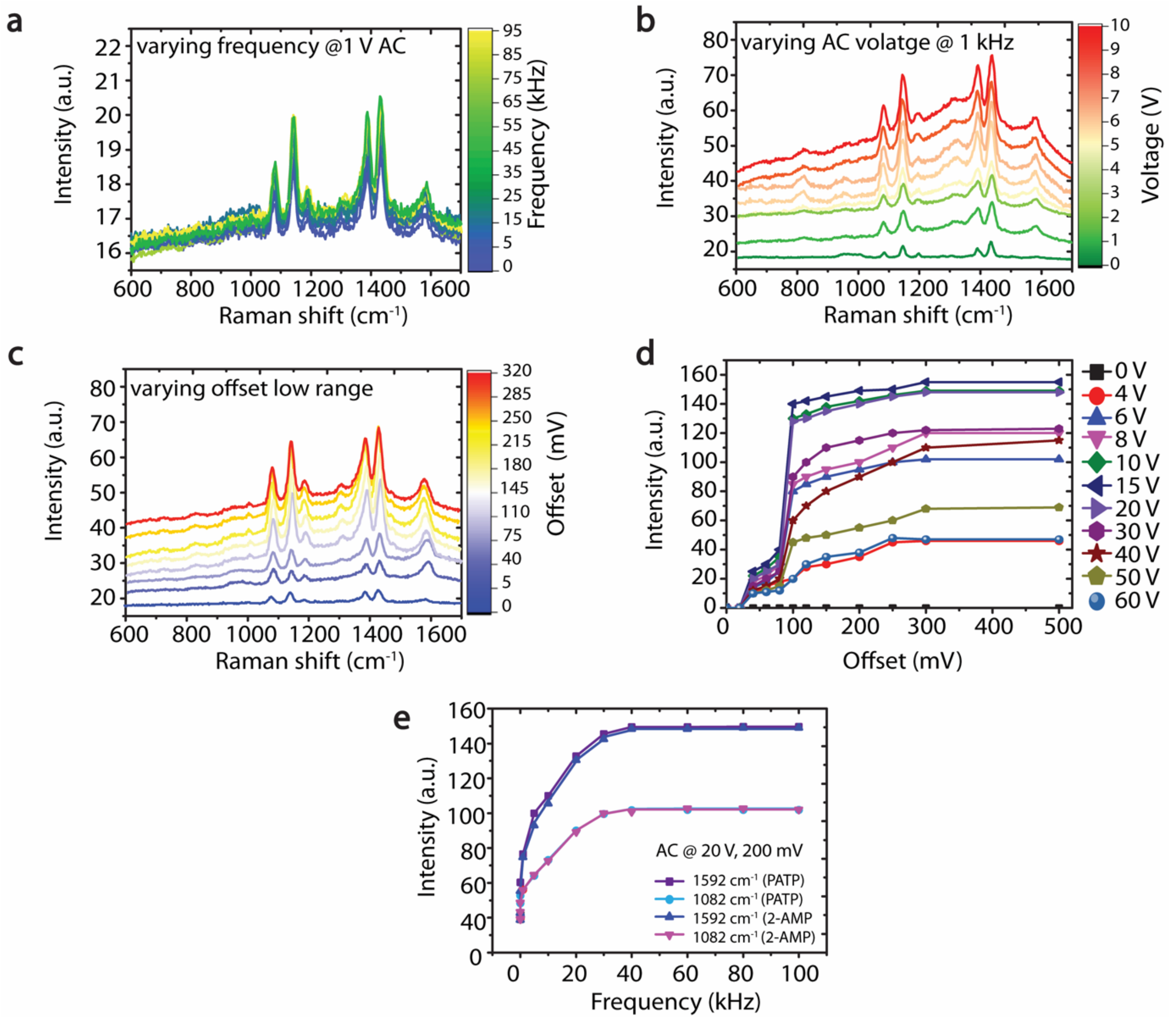

Supplementary Figure 7. SERS measurements of PATP on the FF-PNT/Ag NP template. **(a)** SERS spectra recorded for 1V AC (zero offset) with varying frequency, demonstrating and increase in SERS intensity up to around 40 kHz, after which no further intensity increase is observed. **(b)** SERS spectra at 1 kHz AC, varying voltage (zero offset). **(c)** SERS spectra for varying DC offset bias (0V AC). **(d)** SERS intensity of PNTP band at 1334 cm^{-1} as a function of offset bias at different voltages (40 kHz AC). **(e)** SERS intensity of various bands of PATP and 2-AMP at 20V AC and 200 mV offset bias, as a function of frequency. **(a, b)** demonstrate that at zero offset, no change relative band intensities is observed, and no new Raman bands appear, indicating that the oxidation product PNTP is not generated, independent of AC voltage and frequency. **(c)** demonstrates, through the appearance of new Raman bands, that a threshold offset bias is required for plasmonic catalysis of PATP→ PNTP. **(d)** indicates that the optimum combination of AC voltage and offset is ~15-25 V and >200 mV respectively, while **(e)** shows that >40 kHz AC frequency is required for optimal results (as measured through product yield indicated by product band intensities). Increasing the voltage above ~30V causes a rapid decrease in SERS intensity and catalytic deactivation of the template (the origins of which are both electronic and pyroelectric, as discussed in the main paper. Note that increasing the offset bias >30 V also results in a sharp reduction in SERS intensity (not shown).

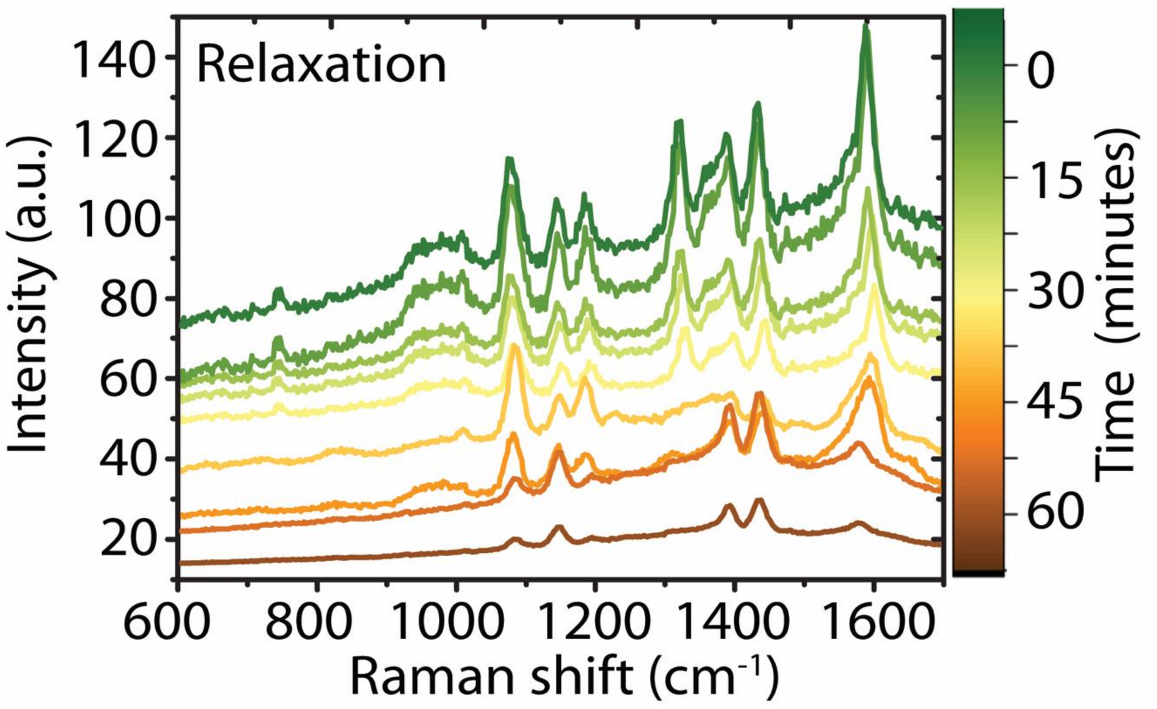

Supplementary Figure 8. SERS spectra of PATP on the FF-PNT/Ag NP template, showing relaxation after removing the electric field. The equilibrated spectrum at t<0 minutes (dark green) is obtained for 10 V AC at 40 kHz with 350 mV offset bias; the electric field is then removed by turning off the electric field (0 V AC and 0 mV offset) at t=0. Spectra are then recorded after t minutes, and demonstrate how the product yield can be controlled. In particular, at long times t>60 minutes, the original spectral features of PATP are recovered. We note also that switching to e.g. 4 V AC with 100 mV offset at t=0 produces very similar results, since these conditions are below the threshold for PNTP generation by template-mediated plasmonic catalysis.

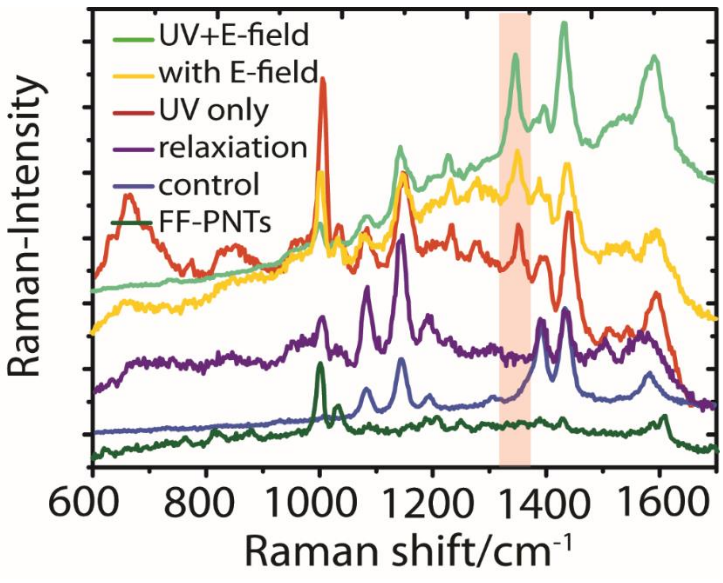

Supplementary Figure 9. SERS spectra of PATP on the FF-PNT/Ag NP template. SERS spectra recorded under different conditions: with electric field (yellow line), with electric field and exposure to UV (light green line), UV exposure only (red line), relaxation after 16 minutes (purple line), control (blue line), and FF-PNTs only (dark green line).

Supporting control studies

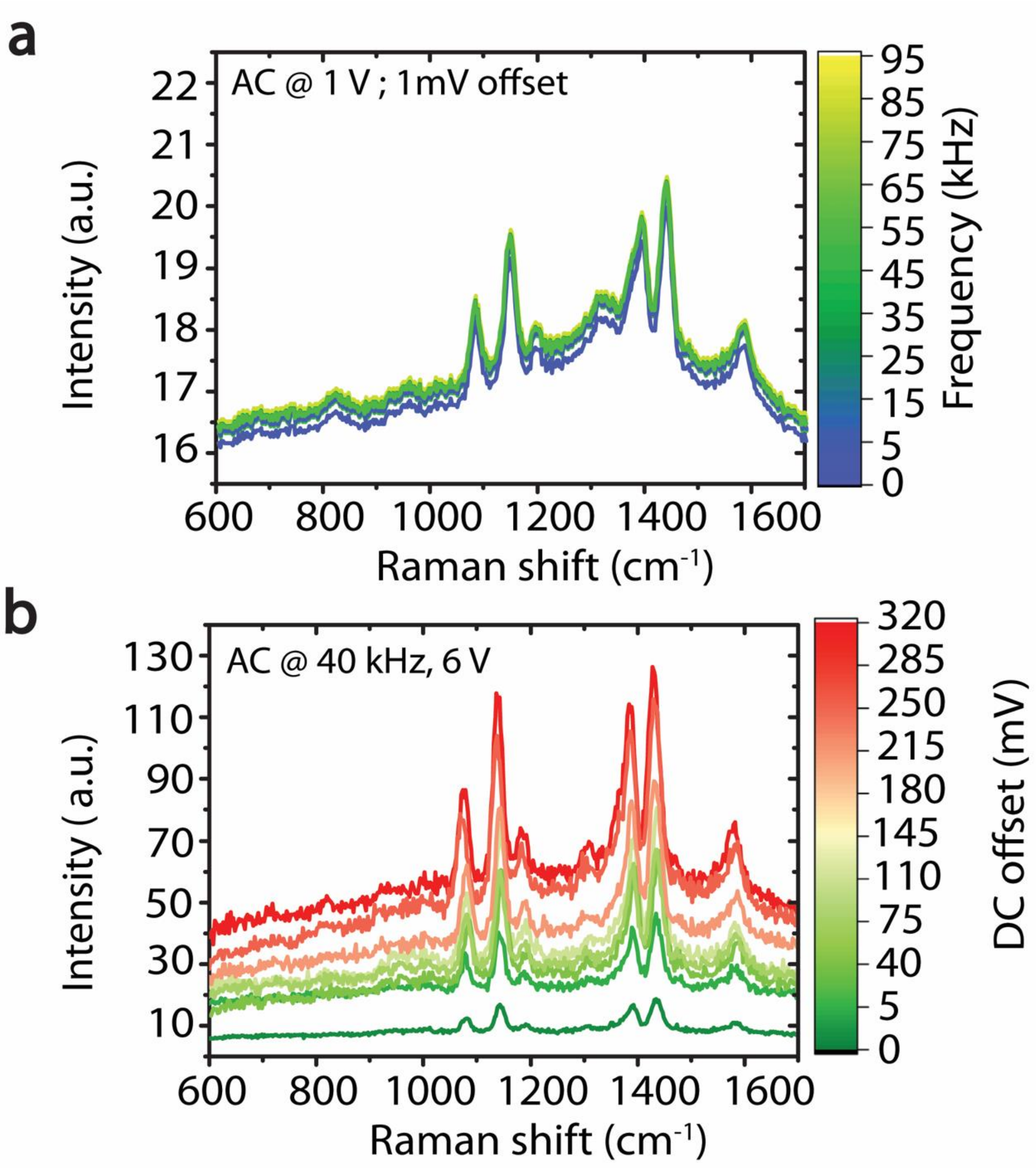

Supplementary Figure 10. SERS spectra of PATP on Ag NPs on a SiO_2 substrate in the absence of FF-PNTs. (a) Constant 1 V AC and 1 mV offset DC bias, varying frequency. Under these conditions, very little change in the SERS spectrum is observed. **(b)** Constant 6V AC at 40 kHz frequency, varying the DC offset bias. Note that, although the SERS signal intensity increases on increasing the offset bias under these conditions, no new bands are observed. This indicates that PNTP is not generated from PATP when using the Ag NPs alone. The template including FF-PNTs is required for plasmonic catalysis of the oxidation reaction; by contrast to the results presented in here **(b),** Fig. 2 shows new bands corresponding to PNTP at e.g. 6 V AC at 40 kHz and 200 mV offset. We speculate that the increase in SERS signal observed here is due to aggregation of Ag NPs forming an electrically conductive network.

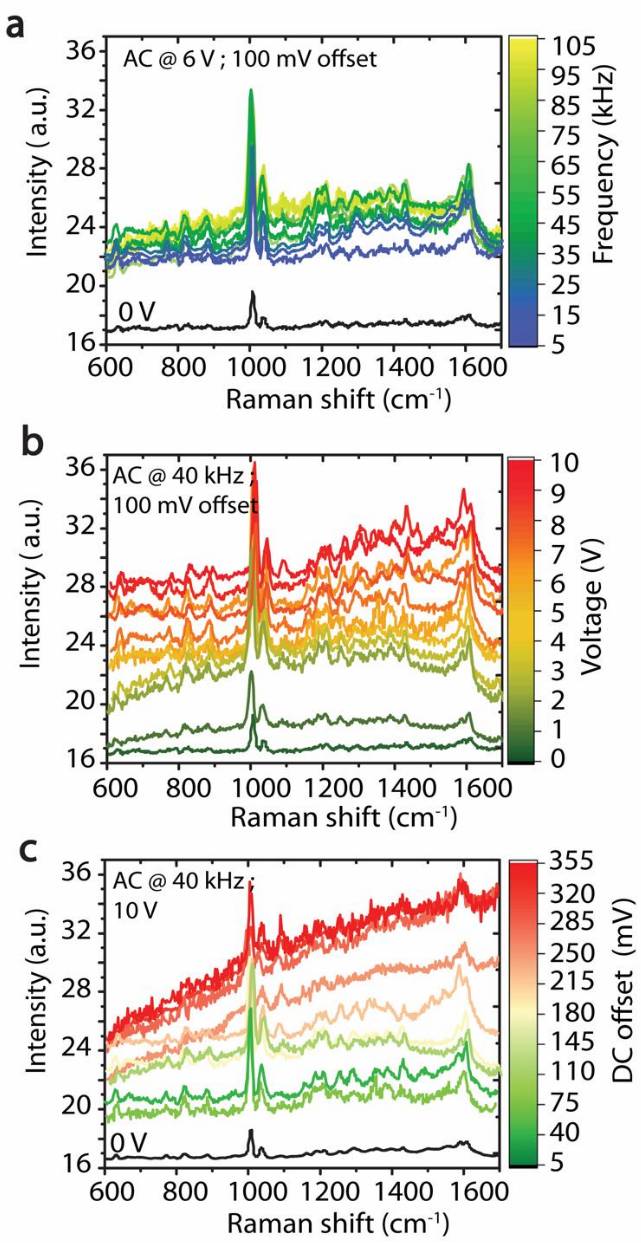

Supplementary Figure 11. SERS measurements of the FF-PNT/Ag NP template (only), in the absence of the probe molecule. (a) Constant 6 V AC with 100 mV bias offset at different frequencies. **(b)** Constant 100 mV bias offset, varying AC voltage (at 40 kHz). **(c)** Constant 10 V AC at 40 kHz, varying DC offset bias. These SERS data, obtained for the template in the absence of the probe molecule, show that all FF-PNTs Raman peaks are clearly visible and detectable under all relevant conditions. In particular, we observe the aromatic ring breathing mode at 1002 cm^{-1} and a phenyl vibrational band at 1603 cm^{-1}. Modes at 1249 cm^{-1} corresponding to amide III vibrations are seen, which indicate a strong coupling between C_α–H and N−H bending vibrations, in agreement with density functional theory calculations reported in the assignment of FF-PNT Raman modes[8–11]. The intensity of the amide I mode (1670 cm^{-1}) can be used to determine the orientation of the carbonyl groups in FF-PN with respect to the nanotube axis. The amide I Raman band is mainly attributed to a C=O stretching vibrational mode along with C–N stretching and C_α–C–N deformation[8–11]. The amide I frequency (1670 cm^{-1}) indicates that only N−H is involved in hydrogen bonding[9,11]. The Raman band at 1418 cm^{-1} (assigned to the symmetric stretching vibration) also showed a strong polarization effect[9,11]. The intense band located at 1002 cm^{-1}, corresponding to the in-plane breathing mode of the phenylalanine benzene rings, increased linearly with applied electric field. Once both the voltage and offset DC bias are increased sufficiently, as shown in **(b)** and **(c),** an increase in SERS intensity was observed, combined with intense fluctuation and blinking in the Raman spectral window between 1200 – 1670 cm^{-1}.

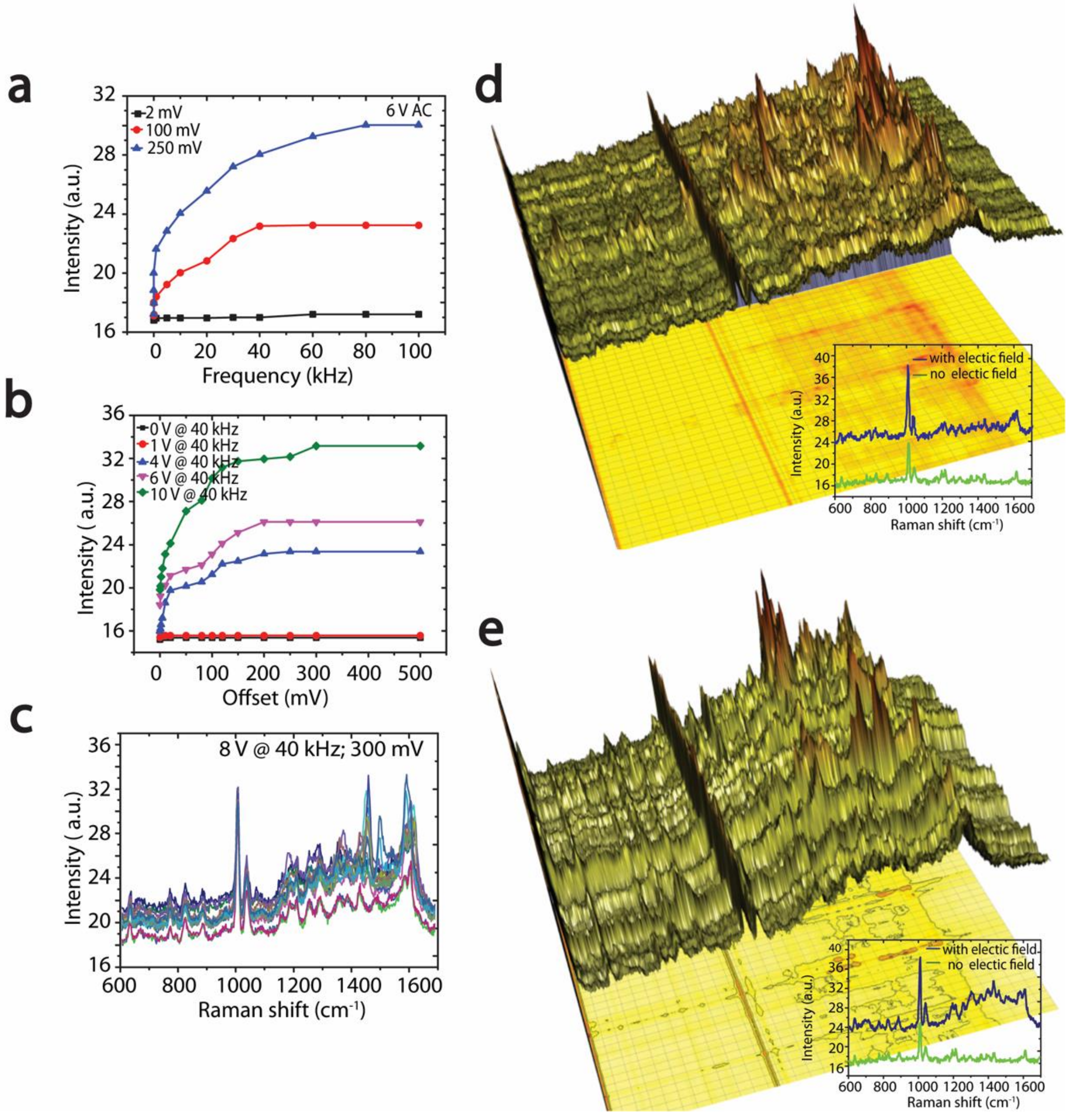

Supplementary Figure 12. SERS spectra of the FF-PNT/Ag NP template (only), in the absence of probe molecule. (a) SERS intensity of the 1002 cm^{-1} band as a function of AC frequency at 6 V for different values of the offset bias. **(b)** SERS intensity of the 1002 cm^{-1} band as a function of offset bias for different voltages, at 40 kHz AC. **(c)** SERS intensity blinking recorded sequentially for 10 V at 40 kHz AC and 300 mV offset. (d, e) 3D time series plots for SERS spectra recorded sequentially for 9 V at 40 kHz AC and 100 mV offset. The strong fluctuation in the spectra could be an indication of possible charge transfer process induced by the electric field. When the AC voltage and bias offset are increased above ~6 V and ~100 mV, respectively, a 4 to 5-fold increase in SERS intensity is observed, combined with intense fluctuation and blinking in the Raman spectral window between 1200 – 1670 cm^{-1}. The blinking observed in the spectra potentially arise from an increase in Ag NP electron density, induced by electric field-activated charge transfer between the FF-PNTs and Ag NPs (see theoretical analysis presented in the main paper). We note there are no signs of geometrical, topological, or morphological changes of the FF-PNT/Ag NP template on application of the electric field, as seen in SEM images (Supplementary Fig.13). These results further establish the stability of the template.

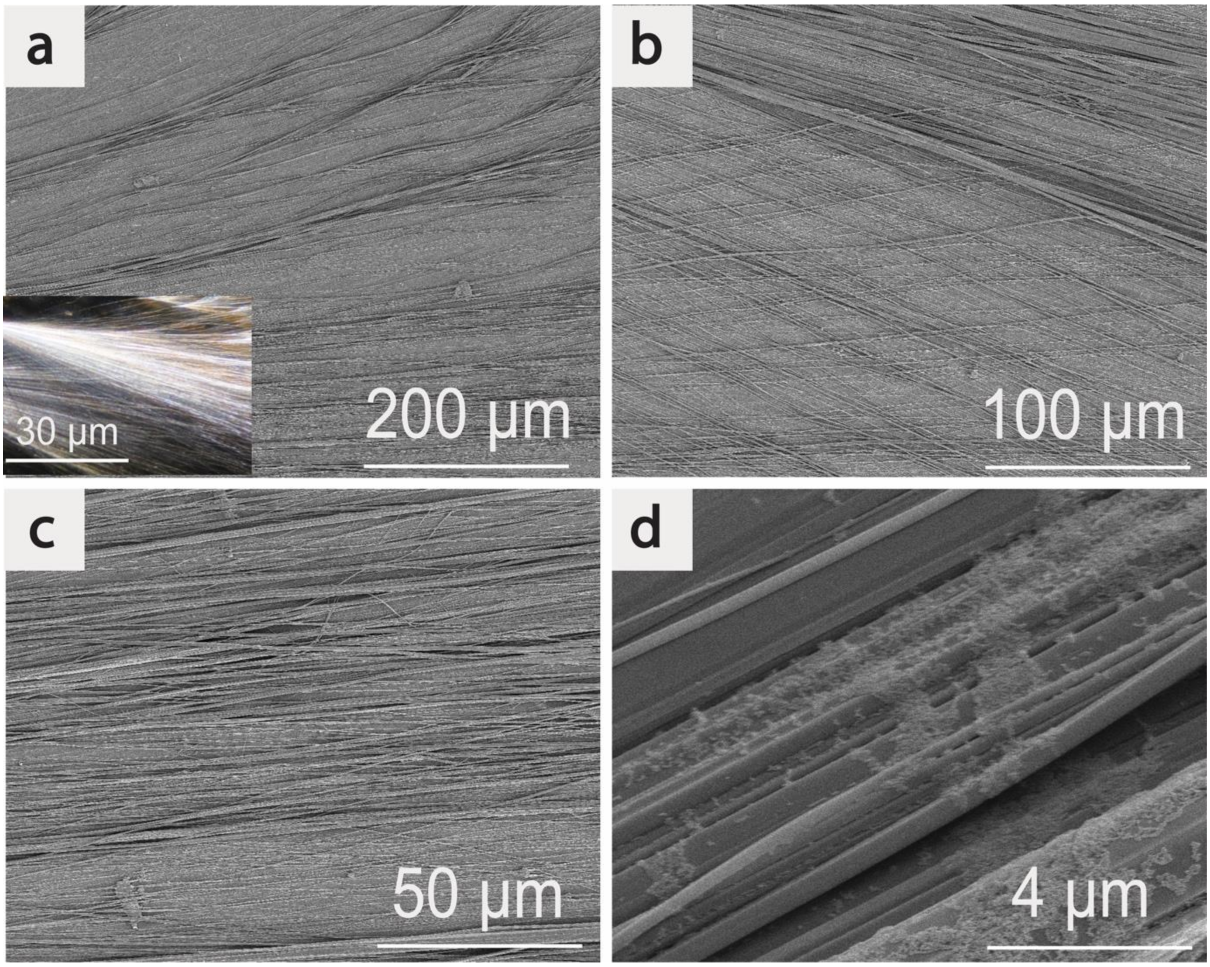

Supplementary Figure 13. Stability of the template after the application of electric field. SEM images of FF-PNTs on the microfabricated device without Ag NPs (a,b) and with Ag NPs (c,d), for 10 V AC at 40 kHz and 300 mV offset. On increasing the voltage to 25 V, no geometrical, topological, or morphological changes are observed, indicating that the template is stable and the structure can withstand application of high voltages, consistent with previous work[10,12], which demonstrated template stability under high laser illumination with a power density of ~6.25 MW/cm^2. These results demonstrate that FF-PNT are promising materials that could be used as an alternative to carbon nanomaterials to prevent photobleaching[10,12].

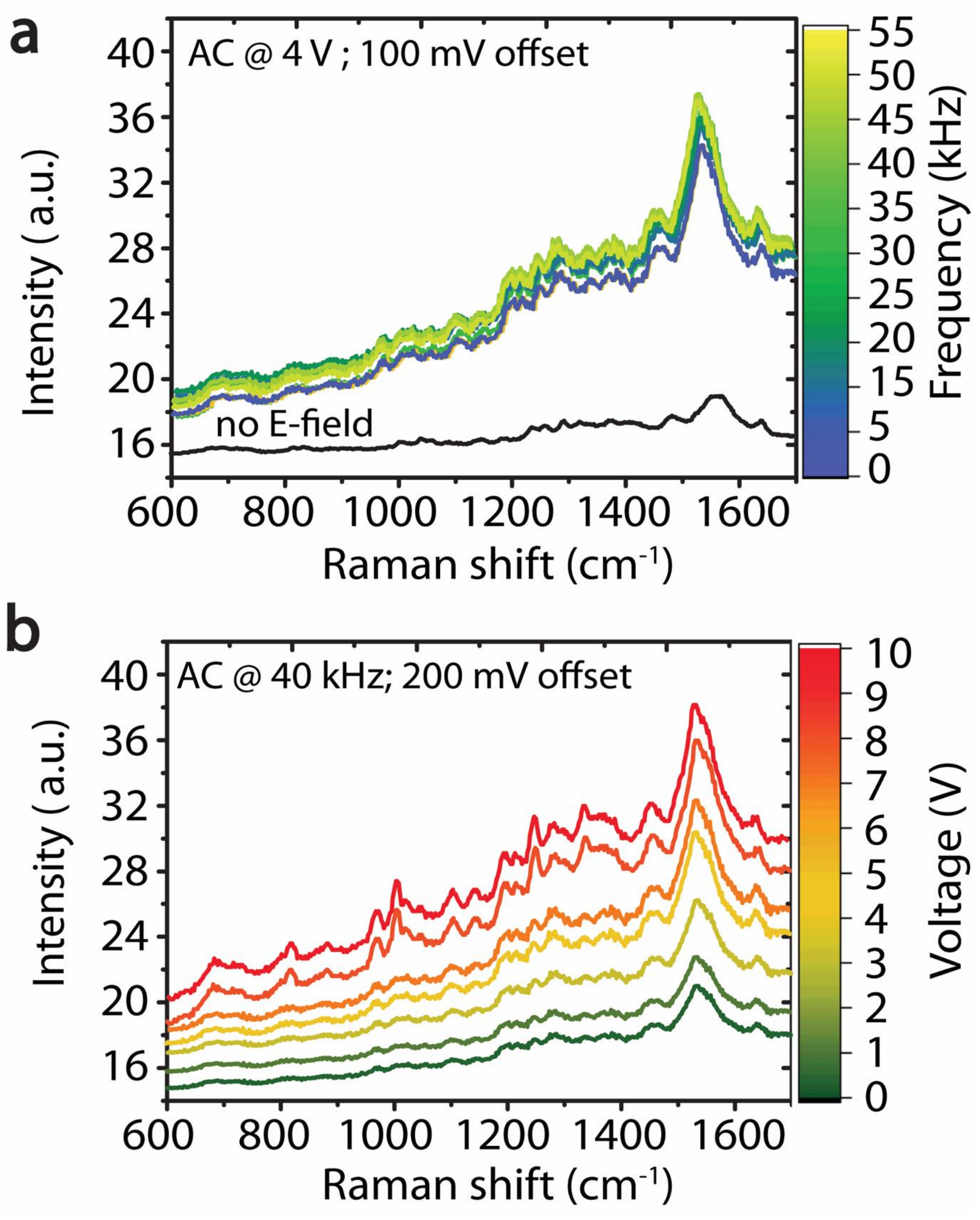

Supplementary Figure 14. SERS measurements of the probe molecule Tetrakis(1-methyl-4-pyridinio) porphyrin (TMPyP) on the FF-PNT template. (a) Constant 4 V AC with 100 mV offset bias, varying frequency (black line shows the zero-field spectrum at 0V for reference). **(b)** Varying voltage at 40 kHz AC, with constant 200 mV offset bias. The intense band at 1552 cm^{-1} exhibits a ~5 fold increase in SERS intensity on increasing voltage to 10 V AC, but no new bands appear.

Optical absorption measurements (UV vis)

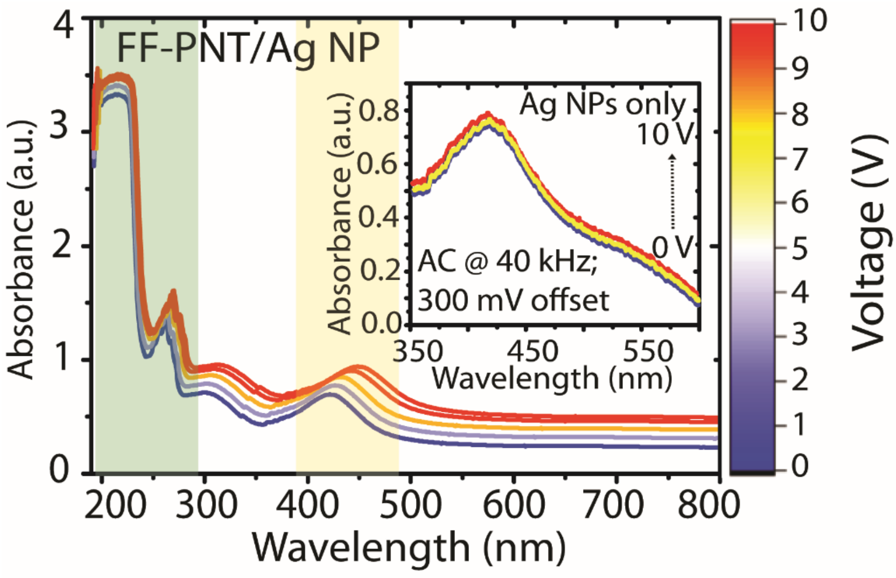

Supplementary Figure 15. UV-vis absorption spectra of the template FF-PNT/Ag NP on glass. UV vis spectra for voltages in the range 1-10 V at 40 kHz AC and 300 mV offset bias. Inset shows for comparison the spectra obtained for Ag NPs only, without the FF-PNTs.

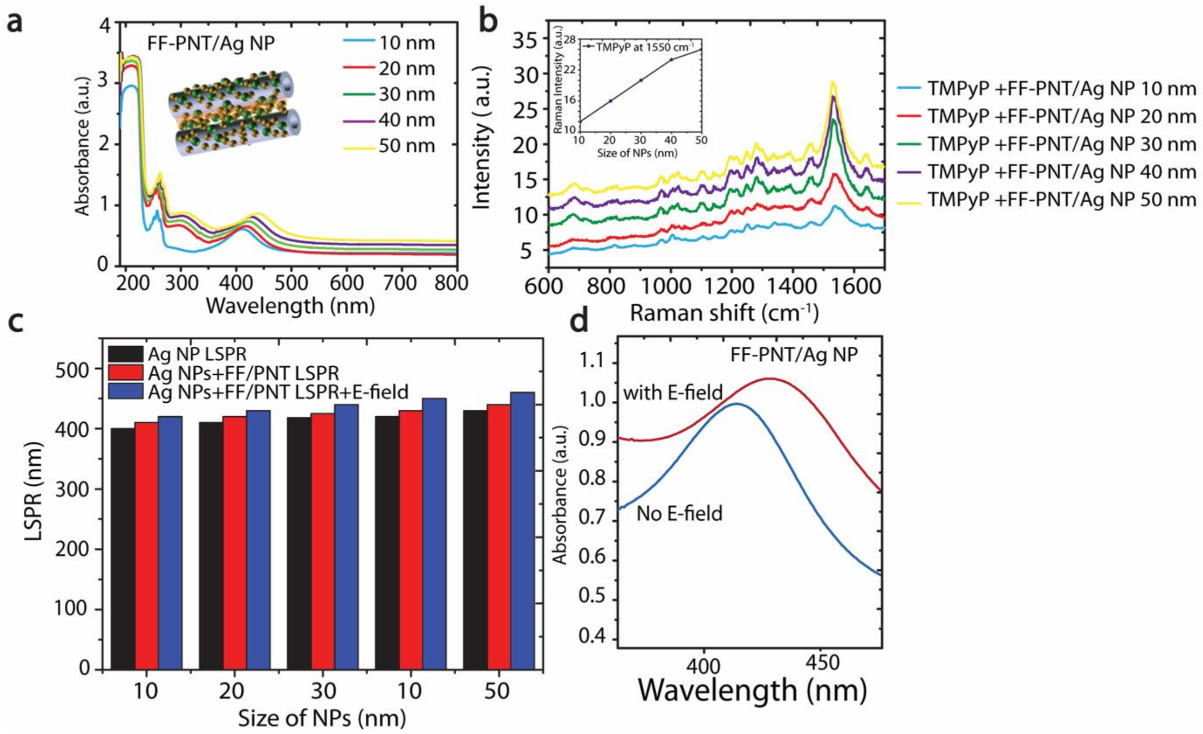

Supplementary Figure 16. **UV vis absorption and SERS spectra of the FF-PNT/Ag NP template for different NP size**. (**a**) UV vis absorption spectra recorded for nanoparticles with different sizes. (**b**) SERS spectra recorded for nanoparticles with different sizes. (**c**) Histogram plot for LSPR frequency as a function of nanoparticle size. (**d**) UV vis absorption spectra recorded with and with an applied electric field.

Larger NP size is found to yield higher SERS intensity and therefore improves sensitivity, consistent with results of previous studies[13]. This is likely due to the higher electromagnetic field associated with larger NPs and the fact that larger NPs aggregate more, leading to more hotspots in the substrate.

Heating effects

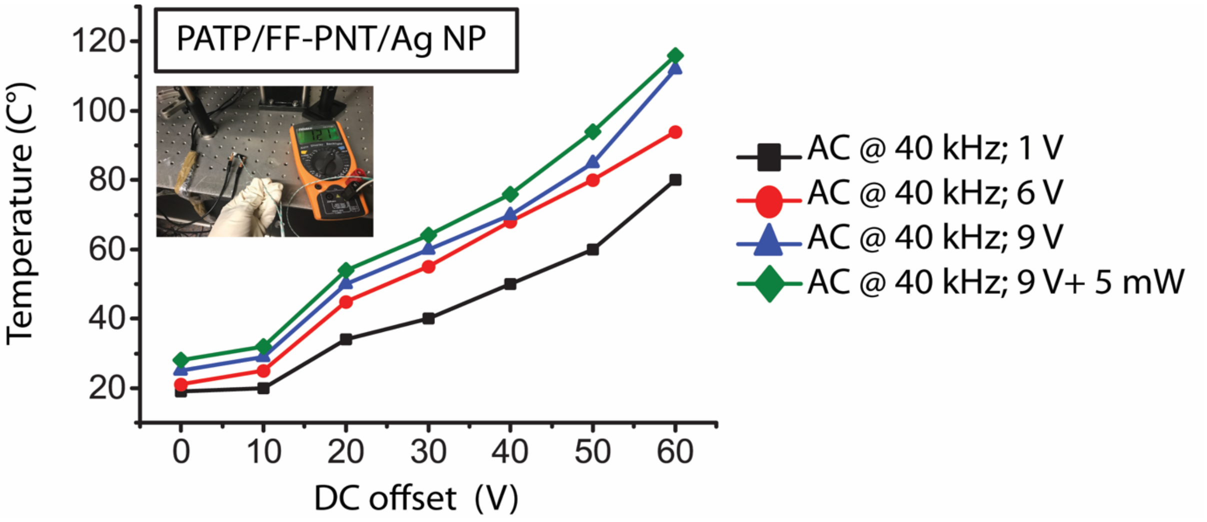

Supplementary Figure 17. **Evolution of temperature of the PATP/FF-PNT/Ag NP system on increasing the DC offset bias, for various AC voltages.** As expected, the temperature increases with both AC and DC voltages. The green points also include the effects of a photoexcitation laser of 5 mW power (λ_{ex} = 532 nm).

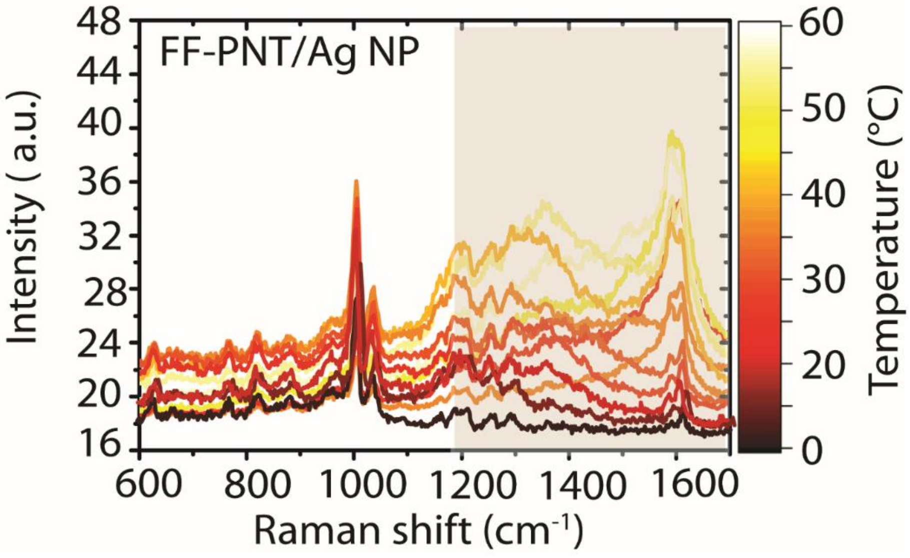

Supplementary Figure 18. SERS spectra of the FF-PNT/Ag NP template at different temperatures. Temperature shown is set and controlled with an electrical hot-plate heater.

Photocatalysis and field effects for molecule 2-AMP

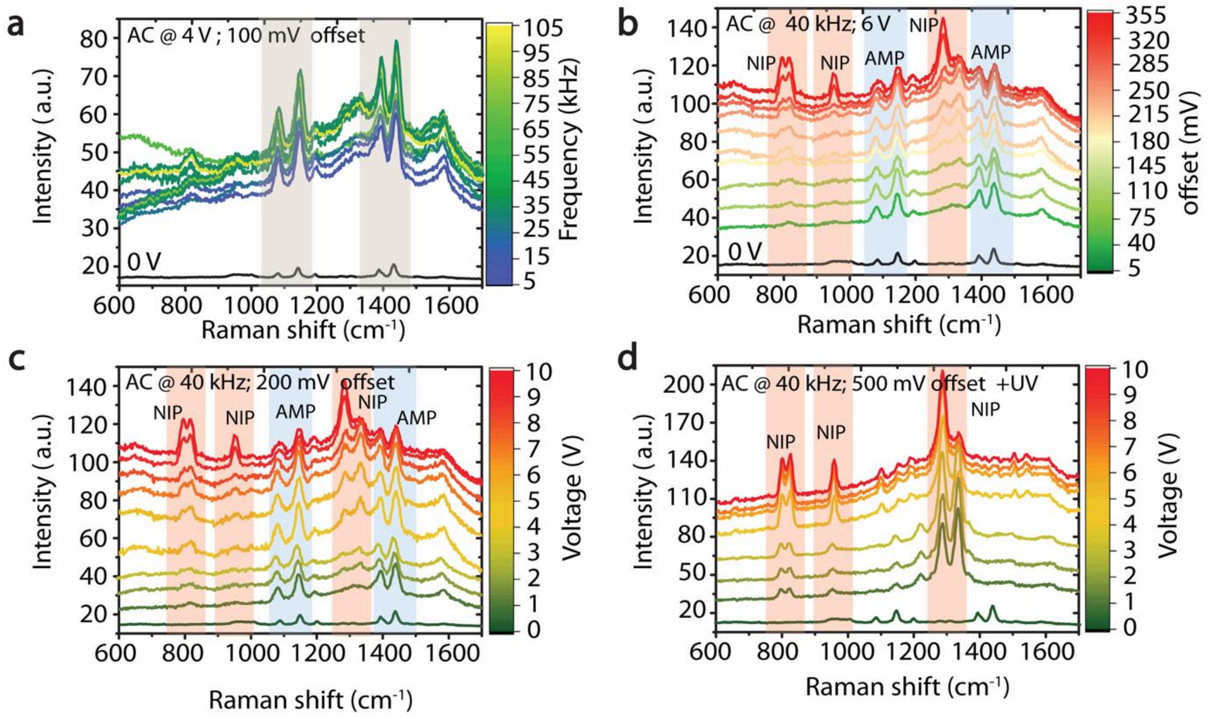

Supplementary Figure 19. SERS spectra of probe molecule 2-AMP on the FF-PNT/Ag NP template under different conditions. (a) Data obtained for constant 4V AC voltage and 100 mV offset bias, varying AC frequency. **(b)** Constant 6V AC at 40 kHz, varying the offset bias. **(c)** Constant 200 mV offset bias, varying AC voltage at fixed 40 kHz frequency. **(d)** As for **(c)** but with 500 mV offset and with additional super bandgap UV irradiation. Without electric field (0V, black reference lines in a, b), the 2-AMP SERS spectra has peaks located at 1580 (C–C symmetric stretching mode), 1440 (C–H in-plane bending modes), 1390 (C–H in-plane bending modes), 1180 (C–H in-plane bending), 1145 (C–N stretching), and 1098 cm^{-1} (CS stretching) [6,7,14–17]. Applying a sufficiently strong electric field (voltage >4 V and offset DC bias >100 mV) results in an ~8 fold increase in SERS intensity (panels b, c). Changes in the SERS spectral features with electric field include the strong appearance of bands at 1285 (C–N stretching mode, n(C–N)) and 1334 (amine scissoring mode, d(NH_2) cm^{-1}), signifying the transformation from 2-AMP to 2-NIP[14–17]. The appearance of bands at lower wave numbers, such as 920 (SH), 858 (C−S stretching mode), and 820 cm^{-1} (CH bending vibration of the nitro group) are also assigned to the oxidized product 2-NIP. Combining both electric field with UV irradiation (λ = 254 nm; for ~20 or 40 min) as in panel **(d)** at different voltages using a high offset DC bias (500 mV) resulted in the rapid transformation of 2-AMP to 2-NIP with a ~10 fold increase in SERS intensity.

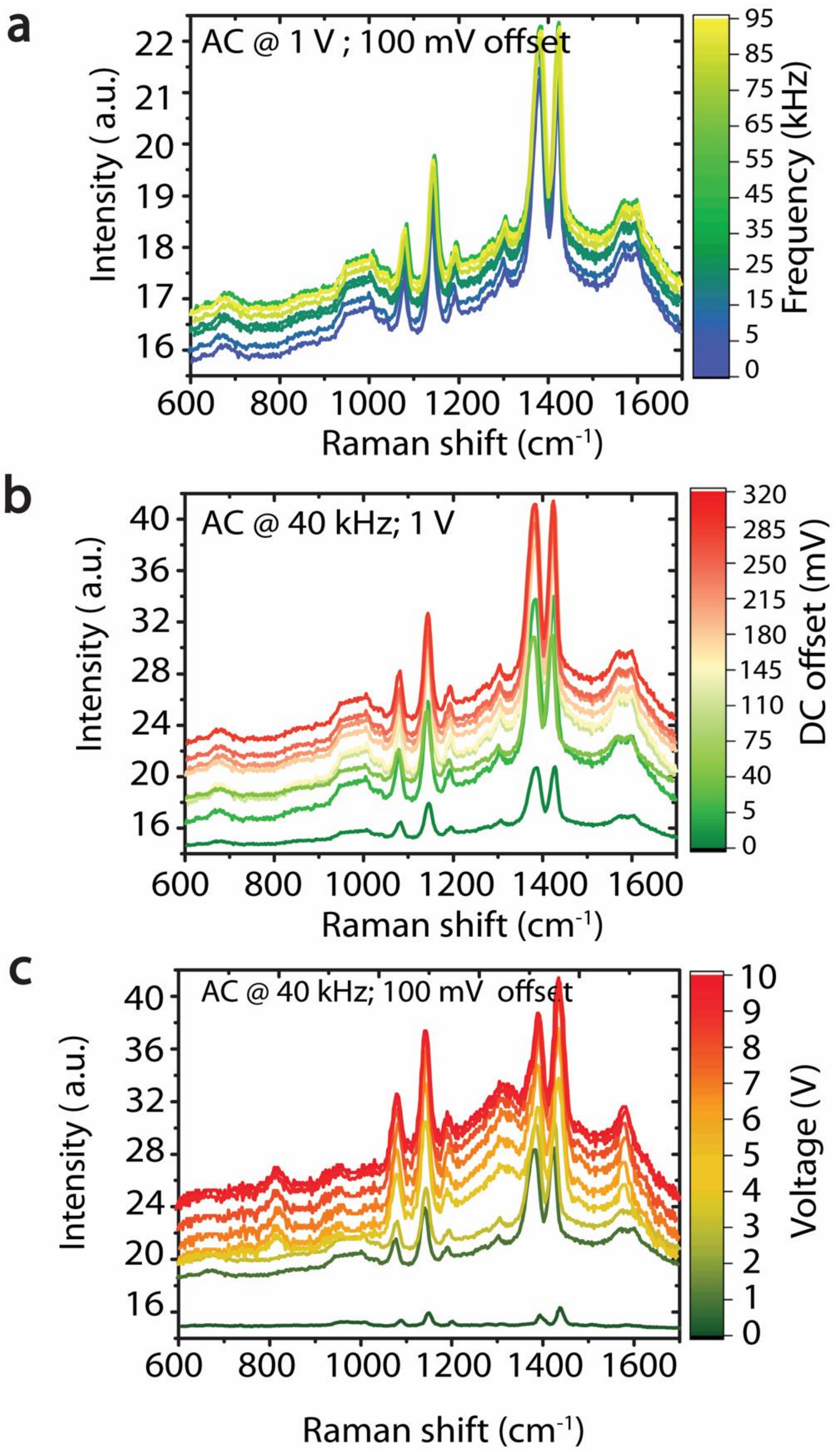

Supplementary Figure 20. **SERS spectra of 2-AMP on Ag NPs on a SiO_2 substrate, in the absence of FF-PNT**. **(a)** 1V AC, with 1mV DC offset bias, varying AC frequency. **(b)** 1V AC at 40 kHz frequency, varying the DC offset bias. **(c)** AC at 40 kHz frequency, varying voltage with constant offset bias of 100mV.

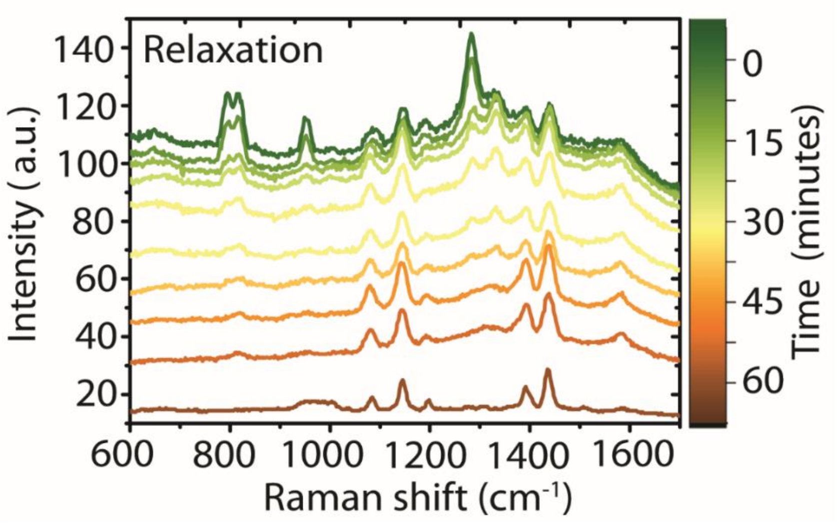

Supplementary Figure 21. **SERS spectra of 2-AMP on the FF-PNT/Ag NP template, showing relaxation after removing the electric field.** The equilibrated spectrum at t<0 minutes (dark green) is obtained for 10 V AC at 40 kHz with 350 mV offset bias; the electric field is then removed by turning off the electric field (0 V AC and 0 mV offset) at t=0. Spectra are then recorded after t minutes, and demonstrate how the product yield can be controlled. In particular, at long times t>60 minutes, the original spectral features of 2-AMP are recovered. We note also that switching to e.g. 4 V AC with 100 mV offset at t=0 produces very similar results, since these conditions are below the threshold for 2-NIP generation by template-mediated plasmonic catalysis.

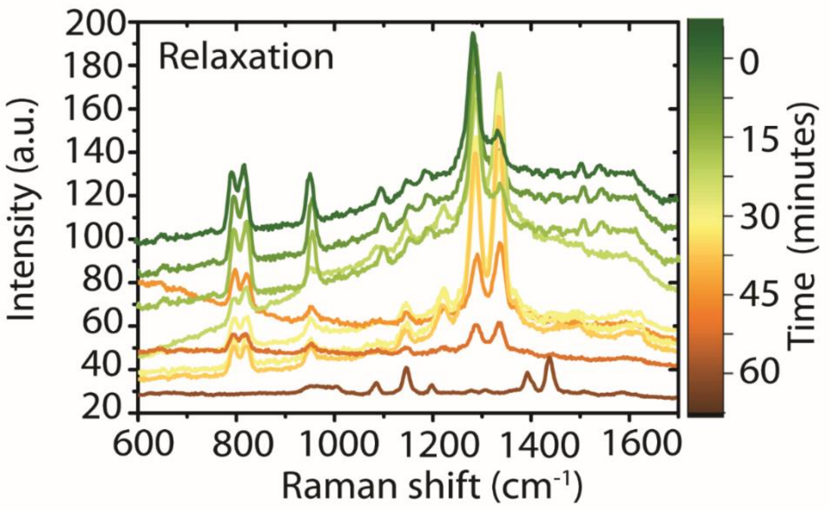

Supplementary Figure 22. SERS spectra of 2-AMP on the FF-PNT/Ag NP template with UV irradiation, showing relaxation. As Supplementary Figure 21, but with additional UV irradiation (λ_{ex} = 532 nm).

SERS sensing

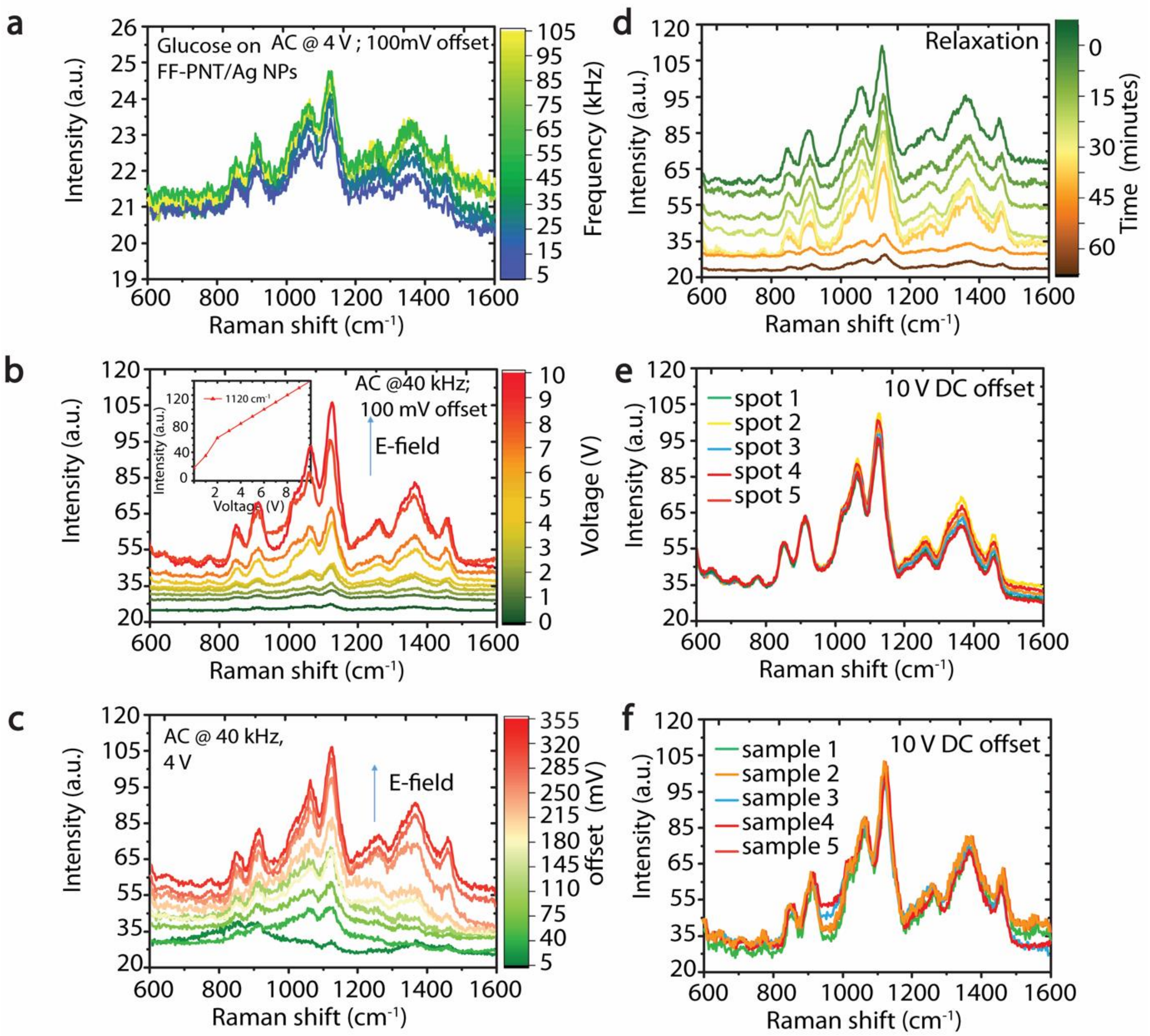

Supplementary Figure 23. SERS measurements of glucose target molecules on the FF-PNT/Ag NP template under different conditions. (a) Constant 4V AC with 100 mV offset bias, varying frequency. **(b)** Constant 100 mV offset bias with 40 kHz AC, varying voltage. Inset shows Raman intensity of the 1120 cm^{-1} band associated with glucose as a function of voltage, showing sensing enhancement. **(c)** Constant 4V AC at 40 kHz, varying the offset DC bias, showing sensing enhancement. **(d)** Relaxation after removing electric field, following the protocol of Supplementary Figure 21. **(e)** Uniformity and reproducibility tests on the sample at different physical locations (spot tests), showing a 5% variability in signal intensity. **(f)** As in **(e)** but with using different samples, and showing a 4% variability in signal intensity.

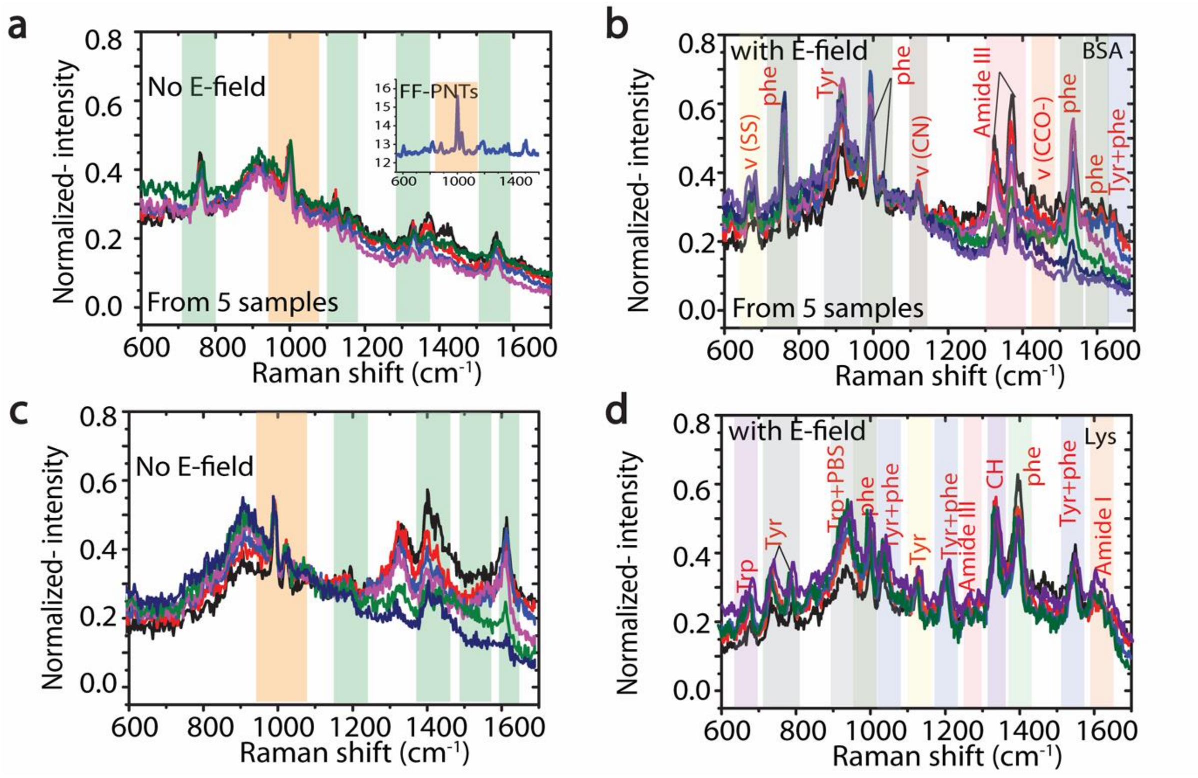

Supplementary Figure 24. Reproducibility tests on the template. SERS measurements of target molecule BSA **(a,b)** and Lys **(c,d)** on the FF-PNT/Ag NP template, without electric field **(a,c)** and with electric field (b,d). The different spectra are for different samples, showing a 5-6% variability in signal intensity, thereby demonstrating the good reproducibility of field-enhanced sensing using the FF-PNT/Ag NP template[21,22].

Supplementary Table 1. Raman peak positions in the SERS spectra of thymine with FF-PNT/Ag NP template. Assignment of the bands are shown based on refs[19,20]. Shown also are band intensities: vs, very strong; s, strong; m, medium; w, weak; vw, very weak, along with conformational assignments: br, broad; bend, bending; breath, breathing; def, deformation; wag, wagging; str, strong.

Wavenumber (cm^{-1})	Plane	Assignment
1600 m	in	$C_2 = O$, $C_4 = O$
1520 m	in	ring str
1477 s	in	bend CH_3
1397 vs	in	bend N_1–H, N_3–H
1352 s	in	bend CH_3, def C_6–H
1279 s	in	ring str
1221 m	in	str C_5–C_9
1198 m	in	bend C_5–H, str C_5–C_6, C_6–N_1
1035 vw	out	wag CH_3
1000 w, 935 s	out	wag N_1–H, N_3–H
820 w	out	ring def
785 vs	in	ring breath
630 w	in	$C_2 = O_2$, $C_4 = O$ def

Supplementary Table 2. Raman shift values in the SERS spectra of uracil with FF-PNT/Ag NP template. Assignment to vibrations of uracil based on refs[19,20]. Shown also are band intensities: vs, very strong; s, strong; m, medium; w, weak; vw, very weak, along with conformational assignments: br, broad; bend, bending; breath, breathing; def, deformation; wag, wagging; str, strong.

Wavenumber (cm^{-1})	Plane	Assignment
1630 s	in	str $C_2 = O$, $C_4 = O$, bend N_1–H, C_5–H
1530 w	in	str C_5–C_6, C_6–N_1, bend C_6–H
1485 m	in	bend N_1–H, C_6–H, C_5–H
1402 vw	in	bend N_1–H, C_6–H, C_5–H
1375 vw	in	bend N_3–H, C_5–H, C_6–H
1300 s	in	bend N_3–H, C_5–H, C_6–H
1279 m	in	str N_3–C_4, C_4–C_5, C_6–N1, bend N_1–H, C_5/6–H
1198 m	in	bend C_5–H, str C_5–C_6, C_6–N_1
1100 m	in	bend C_5–H, str C_5–C_6, C_6–N_1
1020 m	in	ring def
920 vw	out	wag C_6–H
802 w	in	ring breath
780 w<br>700 m	out	ring def
619 w	in	ring def

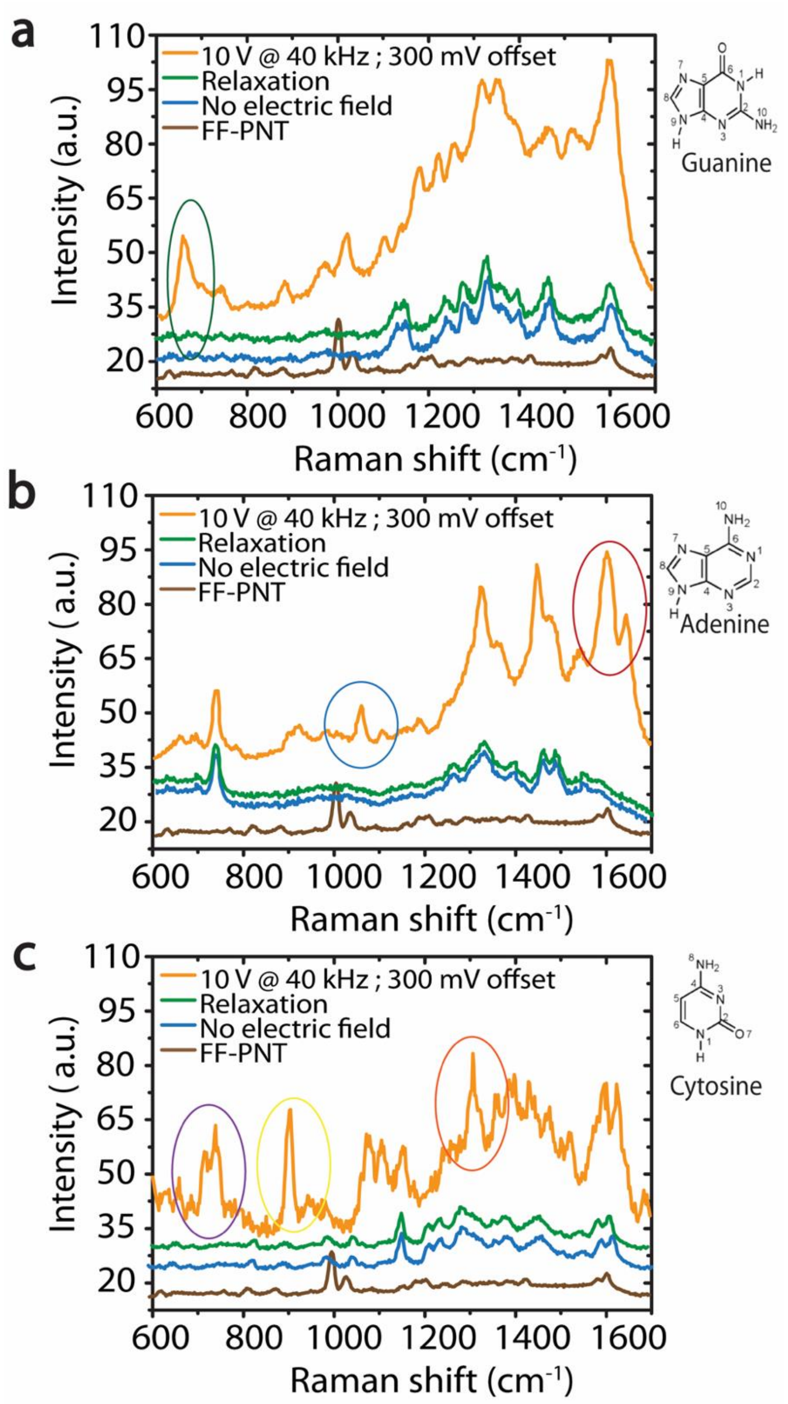

Supplementary Figure 25. SERS sensing from DNA based molecules with electric field. SERS measurements of **(a)** guanine, **(b)** adenine, and **(c)** cytosine, on the FF-PNT/Ag NP template, demonstrating template-mediated field-enhanced sensing. Orange lines correspond to the optimal 10V AC at 40 kHz with 300 mV offset, showing enhancement over the spectra with no electric field (0V, blue lines) or the spectra recorded after relaxation (16 minutes, green lines). The brown lines are for FF-PNT only, without the target molecules, shown for reference. For these molecules, in addition to enhanced sensing, we also observe the appearance of new bands when the electric field is applied, indicating formation of oxidation products from the nucleotides, as highlighted in the figure with colored circles. For example, a new band at 684 cm^{-1} signifies oxidation of guanine[18] in **(a)**. This provides additional evidence of the effect of the electrical field used in conjunction with the template, and its potential utility for plasmonic catalysis.

Supplementary References:

1. Kim, K., Kim, K. L., Lee, H. B. & Shin, K. S. Similarity and Dissimilarity in Surface-Enhanced Raman Scattering of Dimercaptohydrazobenzene on Ag. **116,** 11635–11642 (2012).
2. Sun, M. & Xu, H. Direct visualization of the chemical mechanism in SERRS of 4-aminothiophenol/metal complexes and metal/4-aminothiophenol/metal junctions. *ChemPhysChem.* **10**, 392–399 (2009).
3. Osawa, M., Matsuda, N., Yoshii, K. & Uchida, I. Charge-Transfer Resonance Raman Process in Surface-Enhanced Raman-Scattering From P-Aminothiophenol Adsorbed on Silver - Herzberg-Teller Contribution. *J. Phys. Chem.* **98**, 12702–12707 (1994).
4. Wang, H. Mechanistic understanding of surface plasmon assisted catalysis on a single particle: Cyclic redox of 4-aminothiophenol. *Sci. Rep.* **3,** 2997 (2014).
5. Wang, J.; Ando, R. A.; Camargo, P. H. C. Controlling the Selectivity of the Surface Plasmon Resonance Mediated Oxidation of P-Aminothiophenol on Au Nanoparticles by Charge Transfer from UV-Excited TiO. *Angew. Chemie - Int. Ed.* ***54*****,** 6909–6912 (**2015**).
6. Zhao, L.-B. *et al.* A DFT study on photoinduced surface catalytic coupling reactions on nanostructured silver: selective formation of azobenzene derivatives from para-substituted nitrobenzene and aniline. *Phys. Chem. Chem. Phys.* **14**, 12919 (2012).
7. Jiang, R. *et al.* Photoinduced Surface Catalytic Coupling Reactions of Aminothiophenol Derivatives Investigated by SERS and DFT. *J. Phys. Chem. C.* **120,** 16427–16436 (2016).
8. Almohammed, S. *et al.* Wettability gradient-induced alignment of peptide nanotubes as templates for biosensing applications. *RSC Adv.* **6**, 41809–41815 (2016).
9. Sereda, V., Ralbovsky, N. M., Vasudev, M. C., Naik, R. R. & Lednev, I. K. Polarized raman spectroscopy for determining the orientation of di-d-phenylalanine molecules in a nanotube. *J. Raman Spectrosc.* **47**, 1056–1062 (2016).
10. Almohammed, S., Fedele, S., Rodriguez, B. J. & Rice, J. H. Aligned Diphenylalanine Nanotube-Silver Sanoparticle Templates for High-Sensitivity Surface-Enhanced Raman Scattering. *J. Raman Spectrosc.* **48**, 1799–1807 (2017).
11. Lekprasert, B.; Korolkov, V.; Falamas, A. Investigations of the Supramolecular Structure of Individual Diphenylalanine Nano-and Microtubes by Polarized Raman Microspectroscopy. *Biomacromolecules*. **2012,** 13, 2181–2187 (2012).
12. Almohammed, S., Zhang, F., Rodriguez, B. J. & Rice, J. H. Photo-induced surface-enhanced Raman spectroscopy from a diphenylalanine peptide nanotube-metal nanoparticle template. *Sci. Rep.* **8**, 3880 (2018).
13. Zhou, Y. *et al.* Graphene-silver nanohybrids for ultrasensitive surface enhanced Raman spectroscopy: Size dependence of silver nanoparticles. *J. Mater. Chem. C* **2**, 6850–6858 (2014).
14. Muniz-miranda, M. Application of the SERS Spectroscopy to the Study of Catalytic Reactions by Means of Mono and Bimetallic Nanoparticles. *J. Anal. Bioanal. Tech.* **6**, 6–11 (2015).
15. Hernández-Gordillo, A., Romero, A. G., Tzompantzi, F., Oros-Ruiz, S. & Gomez, R.

Visible Light Photocatalytic Reduction of 4-Nitrophenol using CdS in the Presence of Na2SO3. *J. Photochem. Photobiol. A Chem.* **257,** 44–49 (2013).

16. Aditya, T., Pal, A. & Pal, T. Nitroarene reduction: a trusted model reaction to test nanoparticle catalysts. *Chem. Commun.* **51**, 9410–9431 (2015).
17. Ismail, A. A., Hakki, A. & Bahnemann, D. W. Mesostructure Au/TiO_2 nanocomposites for highly efficient catalytic reduction of p-nitrophenol. *J. Mol. Catal. A Chem.* **358**, 145–151 (2012).
18. Iban, D., Santidrian, A., Heras, A., Kalba, M. & Colina, A. Study of Adenine and Guanine Oxidation Mechanism by Surface- Enhanced Raman Spectroelectrochemistry. *J. Phys. Chem. C*, **119,** 8191–8198 (2015).
19. Ten, G. N., Burova, T. G. & Baranov, V. I. Calculation and Analysis of Vibrational Spectra of Adenine – Thymine, Guanine – Cytosine, and Adenine – Uracil *Mol. BioSyst,* **76,** 84–92 (2009).
20. Madzharova, F., Heiner, Z., Gühlke, M. & Kneipp, J. Surface-Enhanced Hyper-Raman Spectra of Adenine, Guanine, Cytosine, Thymine, and Uracil. *J. Phys. Chem. C* **120,** 15415–15423 (2016).
21. Fazio, B. *et al.* SERS detection of Biomolecules at Physiological pH via aggregation of Gold Nanorods mediated by Optical Forces and Plasmonic Heating. *Sci. Rep.* **6,** 3880 (2016).
22. Boushell, V., Pang, S. & He, L. Aptamer-Based SERS Detection of Lysozyme on a Food-Handling Surface. *J. Food Sci.* **82**, 225–231 (2017).
